# The Impact of Corticosteroid Therapy on Patients With West Nile Virus: A Retrospective Cohort Study

**DOI:** 10.1093/infdis/jiaf601

**Published:** 2025-12-04

**Authors:** Itamar Poran, Bar Basharim, Yaara Leibovici-Weissman, Michal Michaelis, Nassem Ghantous, Noa Eliakim-Raz

**Affiliations:** Internal Medicine E, Rabin Medical Center, Beilinson Campus, Petah-Tikva, Israel; School of Medicine, Faculty of Medical and Health Sciences, Tel-Aviv University, Tel-Aviv, Israel; Internal Medicine E, Rabin Medical Center, Beilinson Campus, Petah-Tikva, Israel; School of Medicine, Faculty of Medical and Health Sciences, Tel-Aviv University, Tel-Aviv, Israel; Internal Medicine E, Rabin Medical Center, Beilinson Campus, Petah-Tikva, Israel; School of Medicine, Faculty of Medical and Health Sciences, Tel-Aviv University, Tel-Aviv, Israel; Internal Medicine E, Rabin Medical Center, Beilinson Campus, Petah-Tikva, Israel; Internal Medicine E, Rabin Medical Center, Beilinson Campus, Petah-Tikva, Israel; School of Medicine, Faculty of Medical and Health Sciences, Tel-Aviv University, Tel-Aviv, Israel; Internal Medicine E, Rabin Medical Center, Beilinson Campus, Petah-Tikva, Israel; School of Medicine, Faculty of Medical and Health Sciences, Tel-Aviv University, Tel-Aviv, Israel; Infectious Disease Unit, Rabin Medical Center, Beilinson Campus, Petah-Tikva, Israel

**Keywords:** corticosteroid, viral encephalitis, West Nile virus, West Nile neuroinvasive disease

## Abstract

**Background:**

West Nile virus (WNV) infection may result in a serious, neuroinvasive, life-threatening disease. Since there is no known therapy against the virus, treatment is based on supportive care. Little is known about the effect of corticosteroids (CS) in patients with West Nile, and their use remains controversial. This study aimed to evaluate the effect of CS treatment in patients with WNV infection.

**Methods:**

A retrospective cohort study was conducted at Rabin Medical Center, including patients with WNV infection. Data were extracted from patients’ electronic medical records. Inverse probability of treatment weighting was used to adjust patient characteristics. Our exposure of interest was CS prescription during the first 48 hours. IPTW-adjusted Cox proportional hazard models were used to compare the risk of hospital mortality. Secondary outcomes included the need for mechanical ventilation or intensive care unit transfer, and the need for rehabilitation or a long-term care facility at discharge.

**Results:**

Data from 150 confirmed cases were extracted. 41 (27%) patients received CSs. After adjusting for potential confounders, CS treatment was found to significantly increase hospital mortality (adjusted hazard ratio [aHR] 3.93, 95% confidence interval [CI] 1.14–13.51). A sensitivity analysis including patients with West Nile neuroinvasive disease and patients hospitalized for more than 48 hours showed consistent results.

**Conclusions:**

Our study suggests that CS use in WNV patients may be associated with an increased risk of hospital mortality, highlighting the need for caution in their use and further prospective investigation.

## Teaser

In a 2024 Israeli cohort of 150 hospitalized West Nile virus patients, early corticosteroid therapy was associated with increased hospital mortality, suggesting potential harm and underscoring the need for caution in corticosteroid use during acute viral infection.

## Graphical Abstract

**Graphical abstract:**
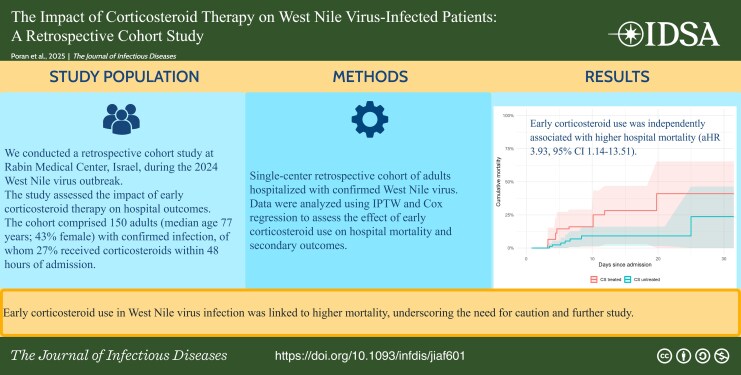
This graphical abstract is also available at Tidbit: https://tidbitapp.io/tidbits/the-impact-of-corticosteroid-therapy-on-west-nile-virus-infected-patients-a-retrospective-cohort-study?utm_campaign=tidbitlinkshare&utm_source=IO

West Nile virus (WNV) is a mosquito-borne RNA virus that primarily circulates among birds. Human transmission is typically through the bite of infected mosquitoes. Following infection, most are asymptomatic. Symptomatic cases are usually self-limited with constitutional symptoms. Approximately 1% of cases progress to West Nile neuroinvasive disease (WNND), characterized by meningitis, encephalitis, and acute flaccid myelitis. In this severe form, the mortality rate can reach up to 10% with considerable morbidity and long-term sequelae described among recovered patients [1, 2]. Due to its wide distribution and tendency to cause sporadic outbreaks, WNV is now considered one of the most common causes of viral encephalitis [2–4].

In 2024, Israel faced one of the largest outbreaks recorded in the region, with more than 930 hospitalizations and 73 deaths attributed to WNV infection [5, 6]. Since no effective treatment has been found against the virus, management is based on primary prevention and supportive care.

Corticosteroid (CS) treatment may be a potential therapeutic approach for WNV infection, aiming to mitigate host-mediated inflammatory damage by reducing secondary inflammation-driven brain injury and decreasing brain edema that contributes to neurological morbidity [7–11].

However, the immunosuppressive effects of CS may facilitate viral replication and potentially worsen outcomes. Evidence on the efficacy of CS therapy in WNV infection remains limited. The objective of this study was to evaluate the association between CS treatment and hospital mortality in patients with WNV during the outbreak of 2024.

## METHODS

### Study Design, Participants, and Data Collection

We conducted a retrospective cohort study of patients with WNV hospitalized at Rabin Medical Center (RMC) during 2024. We included all adult patients (age ≥18 years) admitted with a confirmed diagnosis of WNV.

Patients’ medical records were reviewed, and relevant data were extracted, including sociodemographic characteristics, comorbidities, clinical signs, and laboratory test results available at the time of hospital presentation.

### Case Definitions

WNV diagnosis was defined as a case with clinical signs and symptoms (eg, fever, headache, body aches, joint pains, vomiting, diarrhea, or rash) in addition to positive WNV immunoglobulin M (IgM) and/or positive WNV RNA polymerase chain reaction (PCR) test detected in blood, urine, or cerebrospinal fluid (CSF).

WNND was defined as confirmed WNV infection in addition to clinical or laboratory presentations of encephalitis, meningitis, or acute flaccid paralysis [12].

Immunosuppress state was defined as an individual receiving immunosuppressive medications, including high-dose CS (Prednisone ≥ 20 mg or its equivalent for ≥14 days), cytotoxic chemotherapy agent in the past 6 months, organ transplantation, bone marrow transplantation, or any active hematological malignancy.

### Treatment Exposure

CS treatment was defined as any systemic (oral or intravenous) CS therapy prescribed to a confirmed case for any cause during its first 48 hours of hospitalization.

### Outcomes

Our primary objective was to assess the association between CS treatment and hospital mortality in patients with WNV. Secondary outcomes include the length of hospital stay (LOS), mechanical ventilation or transfer to intensive care unit (ICU), and the need for rehabilitation or a long-term care facility (LTCF) following hospital discharge.

### Statistical Analysis

Patients classified as receiving no treatment were compared to those treated with CS. Continuous variables are presented as median with interquartile range (IQR), while count and percentage describe categorical variables. We compared baseline variables using the Pearson chi-square (χ²) test for categorical variables, and the Mann–Whitney *U*-test for continuous variables, as appropriate. We used the propensity score method to ensure the balance between the treated and untreated groups. The propensity scores were calculated by performing logistic regression, including all measured potential predictors for treatment and outcome available at hospital presentation. We used the inverse probability of treatment weight (IPTW) to adjust for the differences between the groups, which were obtained using the propensity score. The weights for patients who received treatment were set to the inverse of the propensity score, and for those patients who did not receive treatment; the weights were set to the inverse of (1—propensity score). The balance between groups was evaluated using standardized mean differences (SMD), with values <.1 indicating acceptable covariate balance. Propensity score overlap was assessed visually using density plots, and the distribution of stabilized IPTW weights was inspected with histograms to detect extreme weights. The association of treatment with time-related hospital mortality was analyzed using the Cox proportional hazards model weighted by the IPTW and adjusted for known risk factors for poor outcome in WNV (age, male gender, chronic kidney disease [CKD], immunosuppression, and WNND) and covariates associated with the outcome in univariate analysis [13–15]. Model calibration and potential overfitting were assessed by the calibration slope and bootstrap internal validation via 200 bootstrap re-samples. Events per variable ratios were also calculated to evaluate model stability. A two-sided *P* value <.05 was considered to indicate significance. Missing data was treated as missing. All analyses were performed using IBM SPSS Statistics (version 29) and R software (version 4.5.1).

### Sensitivity and Subgroup Analyses

A sensitivity analysis of the primary outcome was performed, including patients hospitalized for at least 48 hours and patients receiving high-dose CS treatment, defined as prescribed CS ≥40 mg per day of prednisone or its equivalent.

Subgroup analysis of the primary outcome was performed, including patients who underwent lumbar puncture and patients classified with WNND.

### Ethical Consideration

This study was approved by the Rabin Medical Center Institutional Review Board (RMC-0473–24). Requirements for informed consent were waived.

## RESULTS

### General Characteristics of the Study Population

In 2024, a total of 177 patients were hospitalized at RMC with the diagnosis of WNV infection. Of those, 27 were excluded: 10 who began CS treatment after 48 hours of admission, 10 with missing clinical or laboratory data, and 7 without definitive virological confirmation of WNV infection. Hence, 150 patients were included in the final analysis (Supplementary Figure 1). The median age was 77 years (IQR 69–84), and 64 patients (43%) were female. Overall, 41 patients (27%) received CS treatment while 109 (73%) did not. Of the treated group, 18 (44%) were under CS treatment before hospitalization, and 14 (34%) were immunosuppressed according to the specified definitions. The most common CS therapy was prednisone (41%), the median daily dose was 40 mg (IQR 18–70) of prednisone or its equivalent, and the median length of therapy was 3 days (IQR 1–5) (Table 1).

**Table 1.** Corticosteroid Treatment

Corticosteroid Treatment	N (%)
No corticosteroid	109 (73%)
Prednisone	17 (11%)
Hydrocortisone	6 (4%)
Dexamethasone	14 (9%)
Methylprednisolone	4 (3%)
Corticosteroid treatment duration, days, median, IQR	3 (1–5)
Corticosteroid dose, prednisone, mg/d, median IQR^a^	40 (18–70)
Total Corticosteroid, prednisone, mg/d, median IQR^a^	125 (67–201)

^a^The median dose of administered steroid treatment, referred to in mg of prednisone.

Compared with the nontreatment group, patients who received CS were more frequently immunosuppressed, had a history of lung disease, and exhibited a higher CHARLSON comorbidity index. In the total cohort, 78 (52%) patients were classified with WNND, 19 (46%) in the CS group, and 59 (54%) in the nontreatment group (Table 2).

**Table 2.** Baseline and Clinical Characteristics at the Time of Presentation According to Treatment Group

	Total	No Treatment	CS Treatment	*P* Value
	N = 150	N = 109	N = 41	
Demographics				
Sex (female)	64 (43)	45 (41)	19 (46)	.57
Age, years	78 (69–84)	78 (70–84)	77 (68–82)	.55
Comorbidities				
Charlson comorbidity index	3 (1–5)	3 (1–4)	4 (2–7)	**.007**
Functional status, dependent	54 (36)	37 (34)	17 (41)	.39
Dementia	30 (20)	24 (22)	6 (15)	.31
Stroke	34 (23)	23 (21)	11 (27)	.45
Lung disease	27 (18)	12 (11)	15 (37)	**<**.**001**
Liver disease	7 (5)	6 (5)	1 (2)	.42
Ischemic heart disease	38 (25)	28 (26)	10 (24)	.87
Diabetes mellitus	65 (43)	49 (45)	16 (39)	.51
Hypertension	96 (63)	69 (63)	26 (63)	.99
Dyslipidemia	82 (55)	59 (54)	23 (56)	.82
Heart failure	25 (17)	15 (14)	10 (24)	.12
Chronic kidney disease	26 (17)	17 (16)	9 (22)	.35
Corticosteroid before admission	19 (13)	1 (0.9)	18 (44)	**<**.**001**
Immunosuppression^a^	21 (14)	7 (6)	14 (34)	**<**.**001**
Organ transplant	9 (6)	0	9 (22)	
Solid cancer	13 (9)	4 (4)	2 (5)	
Hematological malignancy	4 (3)	2 (2)	2 (5)	
Rheumatic diseases	8 (6)	1 (1)	7 (17)	
Vital signs on admission				
Heart rate, BPM	83 (73–94)	81 (73–93)	86 (73–94)	.58
Systolic blood pressure, mmHg	131 (113–148)	130 (113–147)	137 (116–153)	.6
Diastolic blood pressure, mmHg	70 (61–79)	69 (61–78)	72 (63–80)	.49
Temperature, °C	37.6 (36.8–38.7)	37.5 (36.8–38.5)	37.8 (36.7–39.0)	.35
Oxygen saturation, %	96 (94–98)	96 (95–98)	95 (94–97)	.1
Laboratory results				
White blood cells, K/μL	9.0 (7.0–11.8)	9.0 (6.8–11.5)	9.3 (7.8–12.4)	.31
Hemoglobin, g/dL	12.9 (11.8–14.1)	12.9 (12.0–14.1)	13.0 (11.3–13.9)	.53
Platelet, K/μL	185 (146–224)	181 (144–224)	197 (157–235)	.39
Creatinine, mg/dL	1.1 (0.8–1.4)	1.1 (0.8–1.4)	1.2 (0.9–1.5)	.34
Albumin, g/dL	4.1 (3.8–4.3)	4.1 (3.8–4.3)	4.0 (3.8–4.3)	.3
Glucose, mg/dL	126 (108–156)	123 (106–152)	133 (118–168)	.14
CRP, mg/dL	1 (0.3–3.6)	0.8 (0.3–3.1)	1.7 (0.3–4.6)	.2
Lumbar puncture	63 (42%)	47 (43%)	16 (39%)	.65
CSF WBC count, cells/μL	49 (19–170)	47 (16–170)	80 (31–197)	.35
CSF protein, mg/dL	80 (62–110)	80 (61–101)	95 (63–129)	.33
CSF glucose, mg/dL	65 (53–76)	61 (57–76)	73 (50–86)	.9
WNV Laboratory testing				
Serum IgM, n = 101	79 (78)	54 (76)	25 (83)	.41
PCR blood, n = 86	63 (73)	47 (70)	16 (84)	.22
PCR urine, n = 29	18 (62)	15 (60)	3 (75)	.56
PCR CSF, n = 29	19 (65)	15 (62)	4 (80)	.45
CSF IgM, n = 16	16 (100)	9 (100)	7 (100)	N/A
West Nile neuroinvasive disease	78 (52)	59 (54)	19 (46)	.39
Antibiotic treatment first 48 h	103 (69)	72 (66)	31 (76)	.26

Continuous variables are presented as median (IQR), and categorical variables are presented as count (percentage).

CRP, C-reactive protein; PCR, polymerase chain reaction; CSF, cerebrospinal fluid; WBC, white blood cell; N/A, not applicable.

Boldface means statistically significant (*P* < .05).

^a^Numbers do not sum to the total; cases may be represented in more than one group.

**Figure 1.**
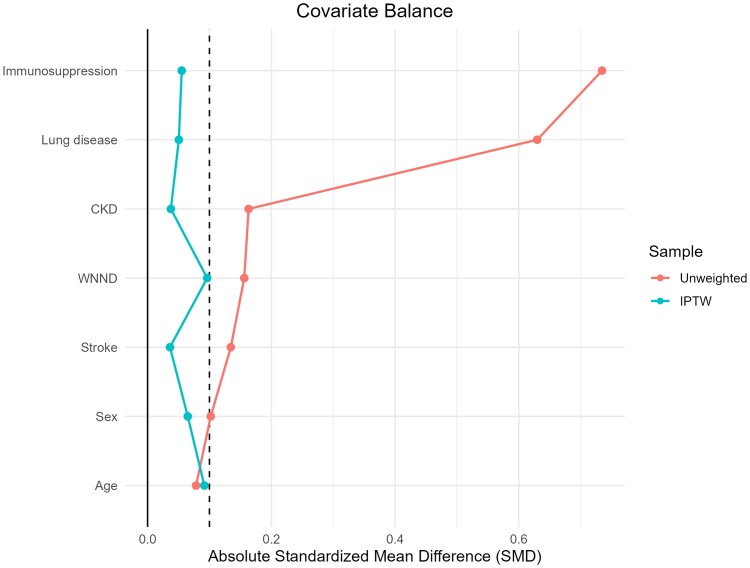
Covariate balance before and after inverse probability of treatment weight. Standardized mean differences (SMD) for covariates before (unweighted) and after IPTW. CKD, chronic kidney disease; WNND, West Nile neuroinvasive disease.

After IPTW, covariates were well balanced (SMD <0.1), the treated and untreated groups exhibited good overlap in the propensity score distribution, and the stabilized weights had a narrow, well-distributed range without extreme values (Figure 1 and Supplementary Figure 2).

### Hospital Mortality

Regarding hospital mortality, a total of 16 (10.6%) patients died during hospitalization: 8 (19.5%) in the CS group and 8 (7.3%) in the nontreatment group. In the unadjusted (crude) analysis, CS treatment was associated with increased hospital mortality (HR 2.94, 95% CI 1.06–8.17, *P* = .038). After adjusting for age, gender, CKD, history of stroke, immunosuppression, and neuroinvasive disease, this association remained significant (adjusted HR 3.64, 95% CI 1.14–11.67, *P* = .03). To further evaluate the effect of CS treatment on hospital mortality, IPTW based on the propensity score was applied. In a doubly robust IPTW-adjusted Cox model, CS treatment remained significantly associated with increased mortality (aHR 3.93, 95% CI 1.14–13.51, *P* = .03). Model discrimination was good (C-index = 0.81), and the calibration plot was approximately 1.0, indicating minimal overfitting (Table 3 and Supplementary Figures 3 and 4).

**Table 3.** Hospitalization Outcomes

Outcome	No Treatment	CS Treatment	HR (95% CI)	*P* Value
	N = 109	N = 41		
Hospital mortality				
Crude	8 (7.3)	8 (19.5)	2.94 (1.06–8.17)	.04
Adjusted^a^			3.64 (1.14–11.67)	.03
Adjusted with IPTW^a^			3.93 (1.14–13.51)	.03
ICU and mechanical ventilation^a^	8 (7.3)	6 (14.6)	4.6 (1.07–19.85)	.04
Rehabilitation or LTCF^a^	26 (25.7)	12 (36.4)	1.1 (0.5–2.36)	.83

IPTW, inverse probability of treatment weight; ICU, intensive care unit; LTCF, long-term care facility.

^a^Adjusted for age, sex, chronic renal failure, immunosuppression, history of stroke, and neuroinvasive disease.

### Secondary Outcomes

Regarding secondary outcomes, the median LOS was similar between the CS-treated and untreated groups (5 vs 6 days, *P* = .9). The need for ICU transfer or mechanical ventilation, after accounting for potential confounders, was higher among the CS group (14.6% vs 7.3%, aHR 4.6; 95% CI 1.07–19.85, *P* = .04). Among the 134 (89%) patients who survived hospitalization, 39 (29%) were discharged for rehabilitation or LTCF, 12 in the CS group and 26 in the no-treatment group (36% vs 26%, *P* = .89) (Table 3).

### Sensitivity and Subgroup Analysis

Given the potential risk of immortal time bias associated with CS treatment initiation, the IPTW-adjusted Cox model was also adjusted to account for treatment initiation (survival time started at day 3); the association between CS treatment and hospital mortality remained (aHR 5.32, 95% CI 1.56–18.12, *P* = .008).

To assess whether the association between CS use and mortality was influenced by dosage, a sensitivity analysis was conducted, categorizing patients into no CS, low-dose, and high-dose groups. Following IPTW-adjusted Cox analysis, only the high-dose group was found to be associated with hospital mortality (aHR 6.44, 95% CI 1.97–21.13, *P* = .002).

**Figure 2.**
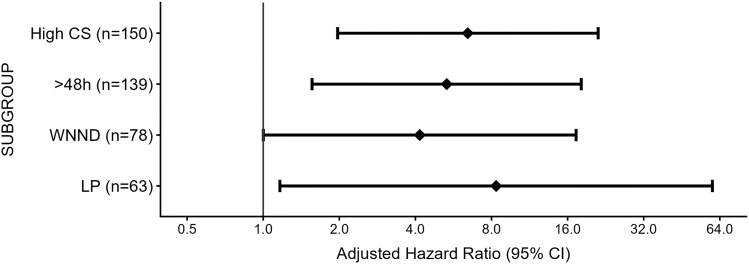
Sensitivity and subgroup analysis. High CS, corticosteroid ≥40 mg/d of prednisone or its equivalent; > 48 h, including patients admitted for more than 48 h; WNND, West Nile neuroinvasive patients; LP, patients who underwent lumbar puncture. Sensitivity and subgroup analyses were adjusted for age, sex, chronic renal failure, immunosuppression, history of stroke, and neuroinvasive disease.

In subgroup analysis, including only patients who underwent lumbar puncture and only patients classified with WNND, CS treatment remains associated with hospital mortality (Figure 2).

## DISCUSSION

In this retrospective cohort study of hospitalized patients with WNV infection during a major outbreak in Israel, early CS therapy was independently associated with a significantly increased risk of hospital mortality. This association persisted after rigorous adjustment for confounding factors and was supported by multiple sensitivity and subgroup analyses. Furthermore, CS use was associated with an increased risk of mechanical ventilation and ICU transfer, though no significant differences were observed in LOS or discharge disposition.

Recent data from Israel's 2024, WNV outbreak underline the importance of recognizing predictors of poor outcomes [5, 16]. Currently, no specific antiviral therapy is approved. Experimental therapies such as IVIG and monoclonal antibodies have not demonstrated clear efficacy [17]. Anti-inflammatory therapies have been tried based on presumed immunopathogenesis; however, management remains largely supportive for now. Current evidence on the use of CS in WNV remains limited, heterogeneous, inconclusive, and mostly derived from observational studies and case series. A previous multicenter retrospective study conducted by Colaneri et al evaluated the effects of CS therapy in 65 patients with WNND across five hospitals in Italy and found that CS treatment did not significantly reduce hospital mortality or neurological sequelae at discharge [18]. Several case reports of WNND patients describe rapid clinical improvement after high-dose CS therapy [8–11]. Systematic review and meta-analysis of a diverse group of viral encephalitis patients have not shown any survival or functional benefit from using CS as an adjunct, and most studies report no significant improvement in outcomes [19]. While our findings align with prior observations that CS therapy does not improve clinical outcomes in WNV, several important differences distinguish our study from earlier reports and deserve mention. First, rather than restricting our cohort to patients with neuroinvasive disease, we examined all hospitalized WNV cases but narrowly defined exposure as CS initiation within the first 48 hours of admission. This approach strengthens the temporal relationship between treatment and outcome and reduces confounding by indication, as late CS use is often triggered by clinical deterioration from secondary complications. Second, unlike previous studies, we applied inverse probability weighting to approximate a causal treatment effect and adjust for baseline imbalances. Taken together, these design and methodological differences may explain the mortality difference and suggest that early CS administration during the viremic phase may have different, and potentially more detrimental, implications than late rescue therapy in critically ill patients with established neuroinvasive diseases.

A possible explanation for our finding is that CS-induced immunosuppression may exacerbate viral replication in the acute phase of WNV infection, potentially leading to worse outcomes. CS broadly suppresses both innate and adaptive immune responses, including inhibition of proinflammatory cytokines, lymphocyte proliferation, and antigen presentation [20]. While this immunosuppressive effect can be beneficial in conditions driven by excessive inflammation, such as autoimmune encephalitis or severe COVID-19, it may be detrimental in the setting of active viral replication, where a robust immune response is required to control viral spread [21]. Animal studies have shown that impaired interferon responses or depletion of immune cells in mice with WNV leads to higher viral loads and increased mortality [22]. Furthermore, CS may hinder the activation of WNV-specific CD8-positive T cells, which are critical for viral clearance in the central nervous system. Suppression of this response may be particularly problematic in WNND [23]. Our data suggests that immune suppression during the early viral replication phase may outweigh any anti-inflammatory benefit. This reinforces the concept that timing of immune modulation is critical, with late-phase inflammation potentially more amenable to immunosuppression, whereas early phase suppression may interfere with viral clearance.

Another key finding is the dose-response relationship seen between high-dose CS treatment and hospital mortality. This suggests that not only the use but also the intensity of CS therapy may affect outcomes, supporting the biological plausibility of CS related harm in WNV infection. Such a relationship could help establish future clinical thresholds for what is considered “high-risk” CS exposure in this context.

Our study has several limitations. First, our observational, retrospective design may result in residual confounding and preclude conclusions about causality. Second, it was a single-center study with a relatively small sample size, which may limit its generalizability. Third, the reliance on hospital records could introduce biases related to documentation practices or missing data. Fourth, it is probable that this outbreak led to over-testing, such that milder cases were more likely to be included in the present analysis. Finally, although the model showed good calibration and discrimination, the low number of deaths raises concern for overfitting. The calibration slope and bootstrap-corrected plots indicate minimal optimism, but the results should be interpreted cautiously and confirmed in larger cohorts.

In conclusion, our study suggests that CS use in patients with WNV may be associated with an increased risk of hospital mortality. In real-world settings, CS are often administered empirically in patients presenting with encephalitis or sepsis-like syndromes before a clear diagnosis is established. Our findings highlight the potential risks associated with this approach in the context of WNV and suggest that a careful diagnostic workup should precede such therapy whenever possible.

## Supplementary Material

jiaf601_Supplementary_Data

## Supplementary Data

Supplementary materials are available at *The Journal of Infectious Diseases* online (http://jid.oxfordjournals.org/). Supplementary materials consist of data provided by the author that are published to benefit the reader. The posted materials are not copyedited. The contents of all supplementary data are the sole responsibility of the authors. Questions or messages regarding errors should be addressed to the author.
