# Supplementary material for: The Impact of Corticosteroid Therapy on Patients With West Nile Virus: A Retrospective Cohort Study
Source: J Infect Dis. 2025 Dec 4;233(3):e667–73. doi: 10.1093/infdis/jiaf601 (PMC13017204; doi:10.1093/infdis/jiaf601)
Supplement: jiaf601_Supplementary_Data [file jiaf601_supplementary_data.zip › SupplementaryMaterial1.docx]

Supplementary Material

**Figure 1.** Patient Flow Diagram

Patients diagnosed with WNV (n=177)

(n = …)

Excluded (n=27)

- 10 CS after 48 hours
- 5 Missing data
- 3 Transferred from another hospital
- 2 Missing insurance number
- 7 Inconclusive serology/PCR results

Patients included (n=150) =150) …)

- Mortality (n=8)
- Hospitalization (6 days)
- Rehabilitation or LTCF (n=26)
- Mortality (n =8)
- Hospitalization (5 days)
- Rehabilitation or LTCF (n=12)

CS treatment (n=41)

- West Nile neuroinvasive disease (n=19)

No treatment (n=109)

- West Nile neuroinvasive disease (n=59)

**Figure 2.** Calibration Plot of the Adjusted IPTW Cox Model for Hospital Mortality.


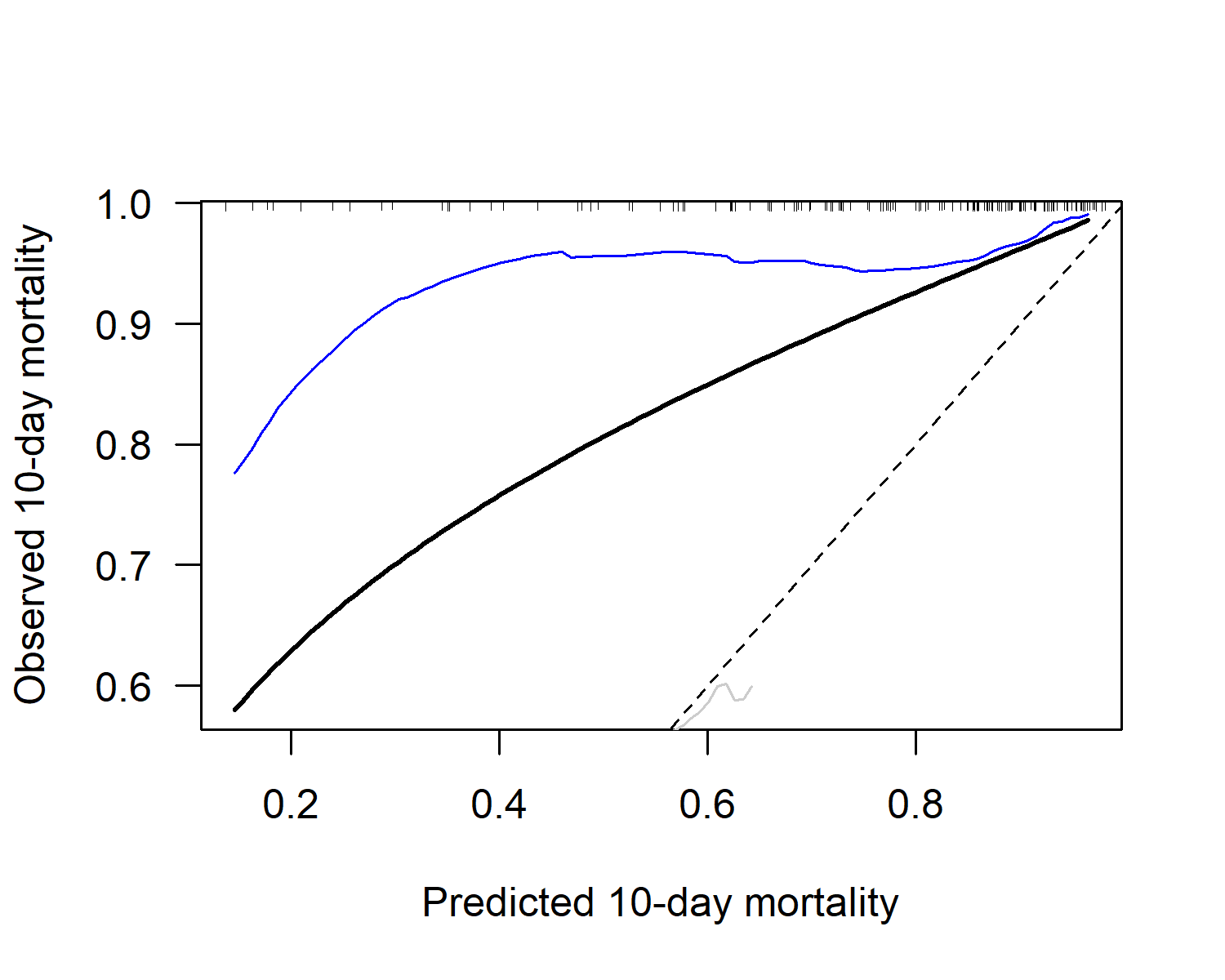


Calibration plot of the adjusted IPTW-weighted Cox model for 10-day hospital mortality.

The black line shows apparent performance, the blue line the bootstrap-corrected performance,

and the dashed line the ideal calibration.

**Figure 3.** Propensity Score Diagnostics Before and After IPTW.


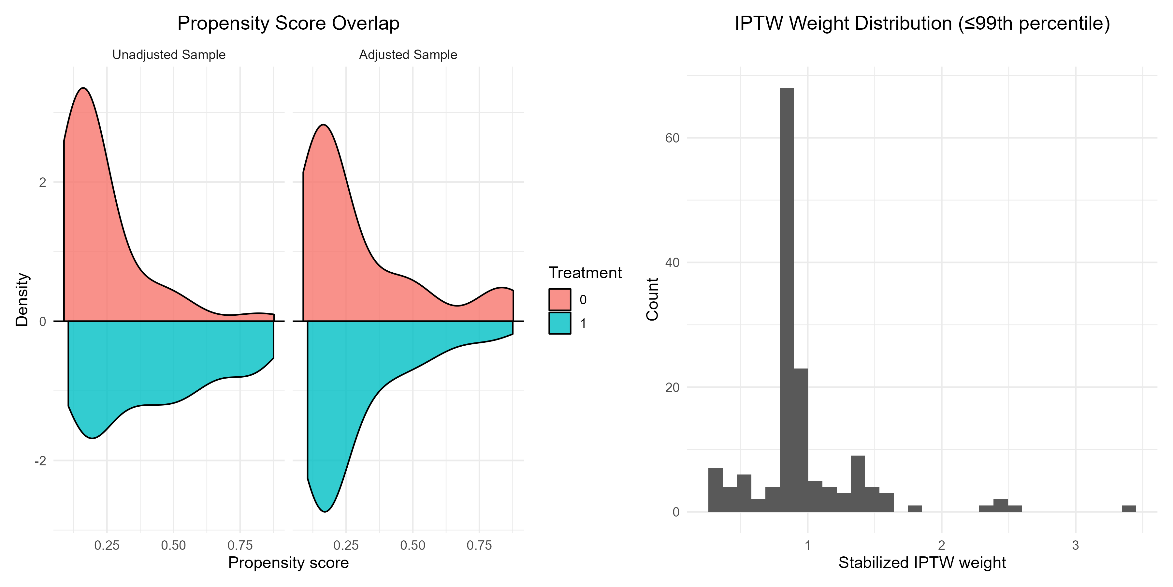


Left: Overlap of propensity scores before and after IPTW.

Right: Distribution of stabilized IPTW weights (trimmed at 99th percentile).

**Figure 4.** IPTW Adjusted Cumulative Mortality Curves for Corticosteroid-Treated and Untreated Patients.


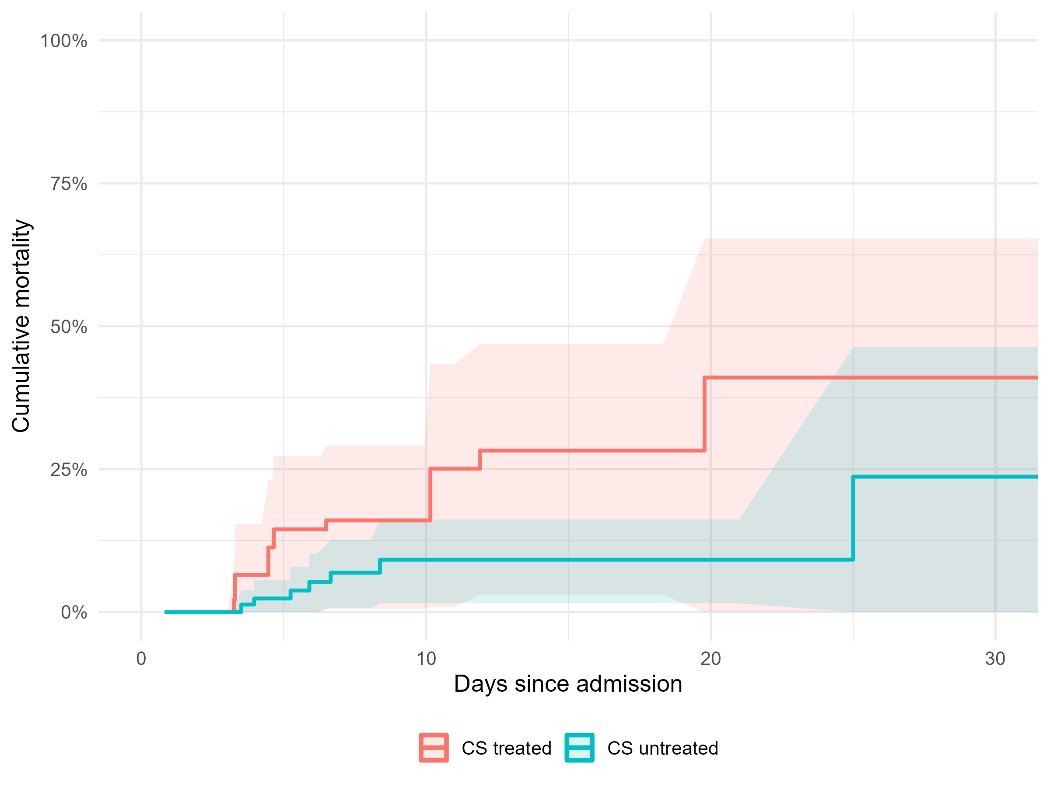


Cumulative mortality curves using IPTW-adjusted Kaplan-Meier analysis. Shaded areas represent 95% confidence intervals (aHR 3.93, 95% CI 1.14-13.51, p = 0.03).
