# Supplementary figures and images for: The Impact of Corticosteroid Therapy on Patients With West Nile Virus: A Retrospective Cohort Study
Source: J Infect Dis. 2025 Dec 4;233(3):e667–73. doi: 10.1093/infdis/jiaf601 (PMC13017204; doi:10.1093/infdis/jiaf601)

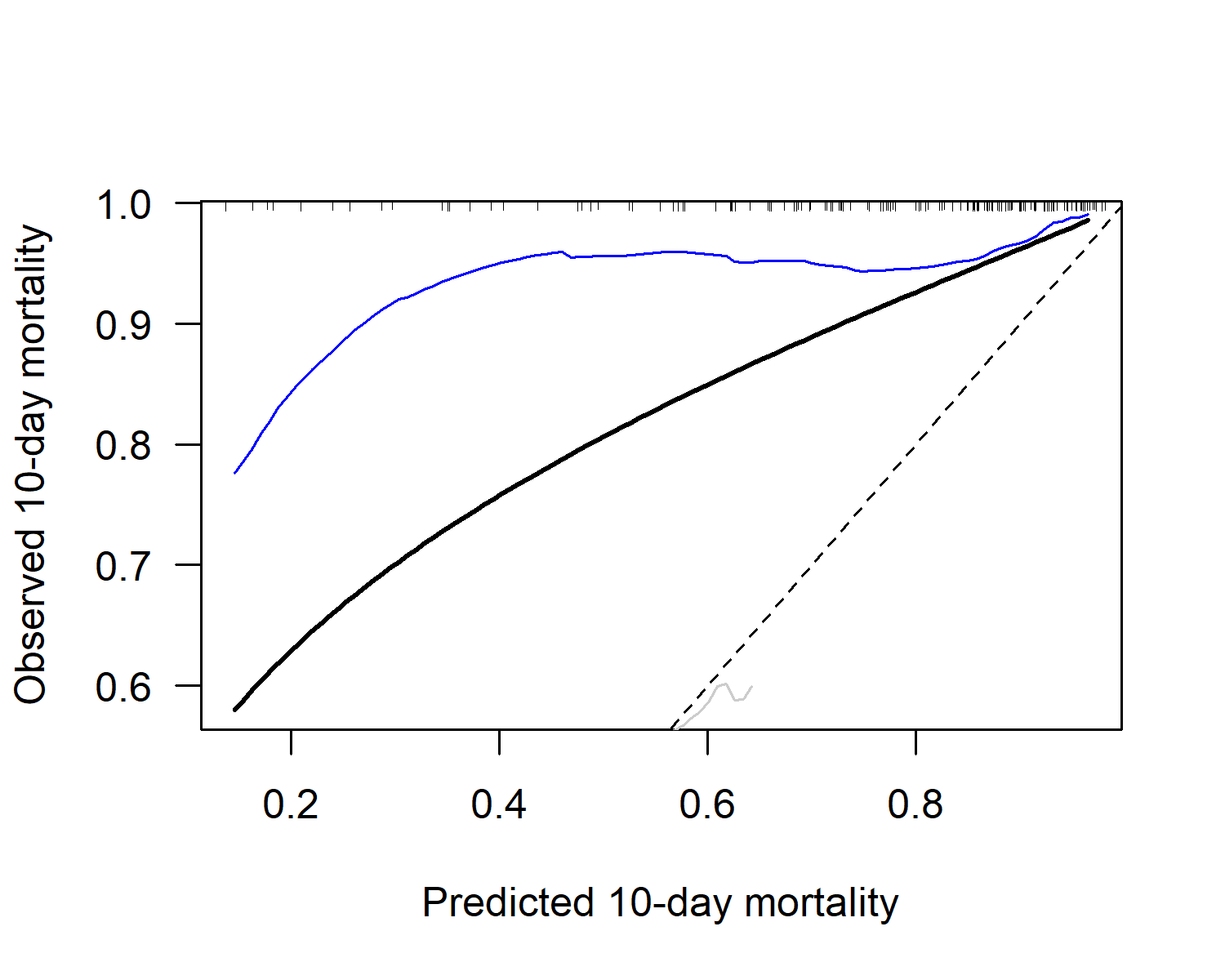

Supplement: jiaf601_Supplementary_Data [file jiaf601_supplementary_data.zip › SupplementaryFigure2.tif]

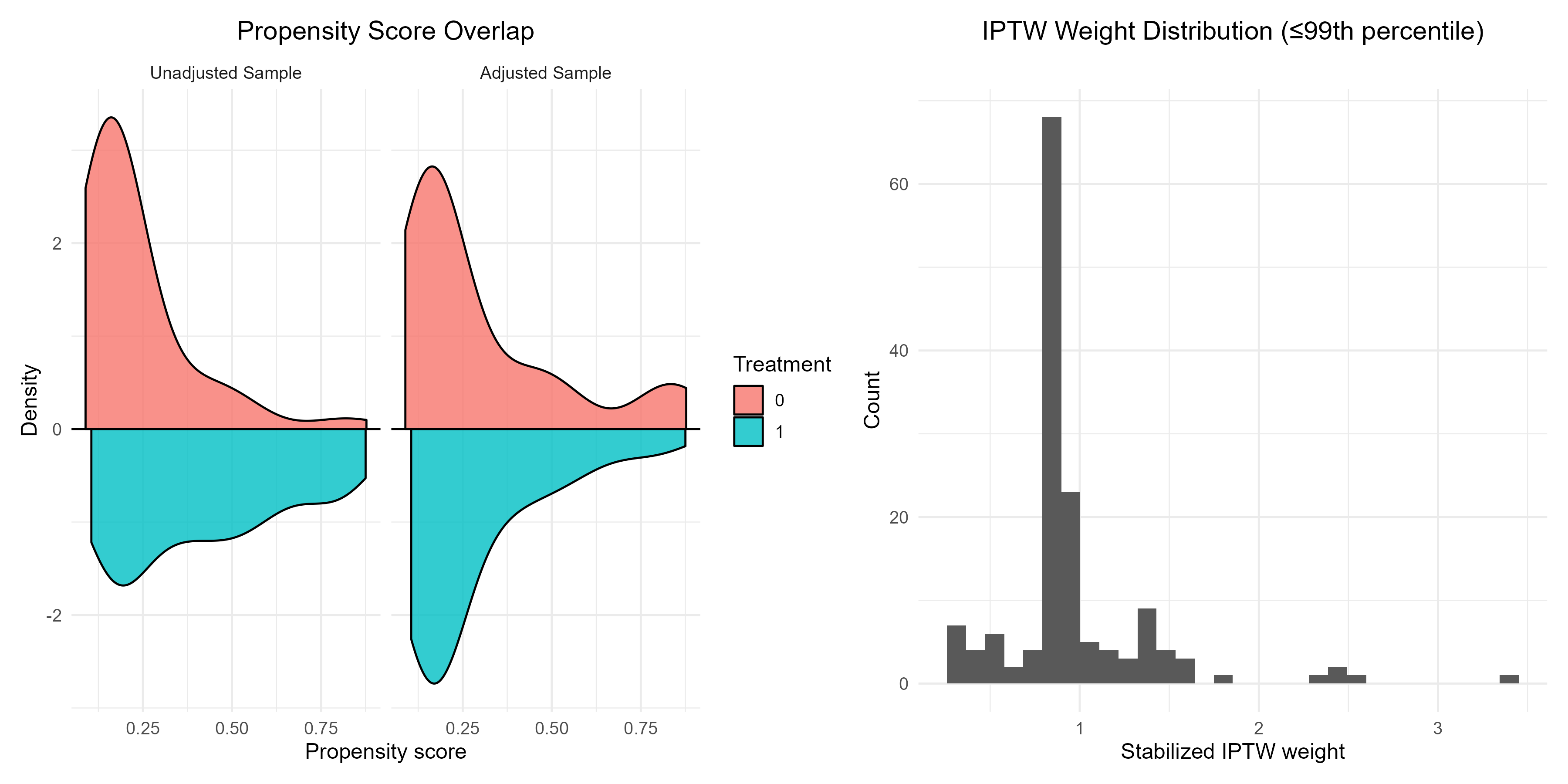

Supplement: jiaf601_Supplementary_Data [file jiaf601_supplementary_data.zip › SupplementaryFigure3.tif]

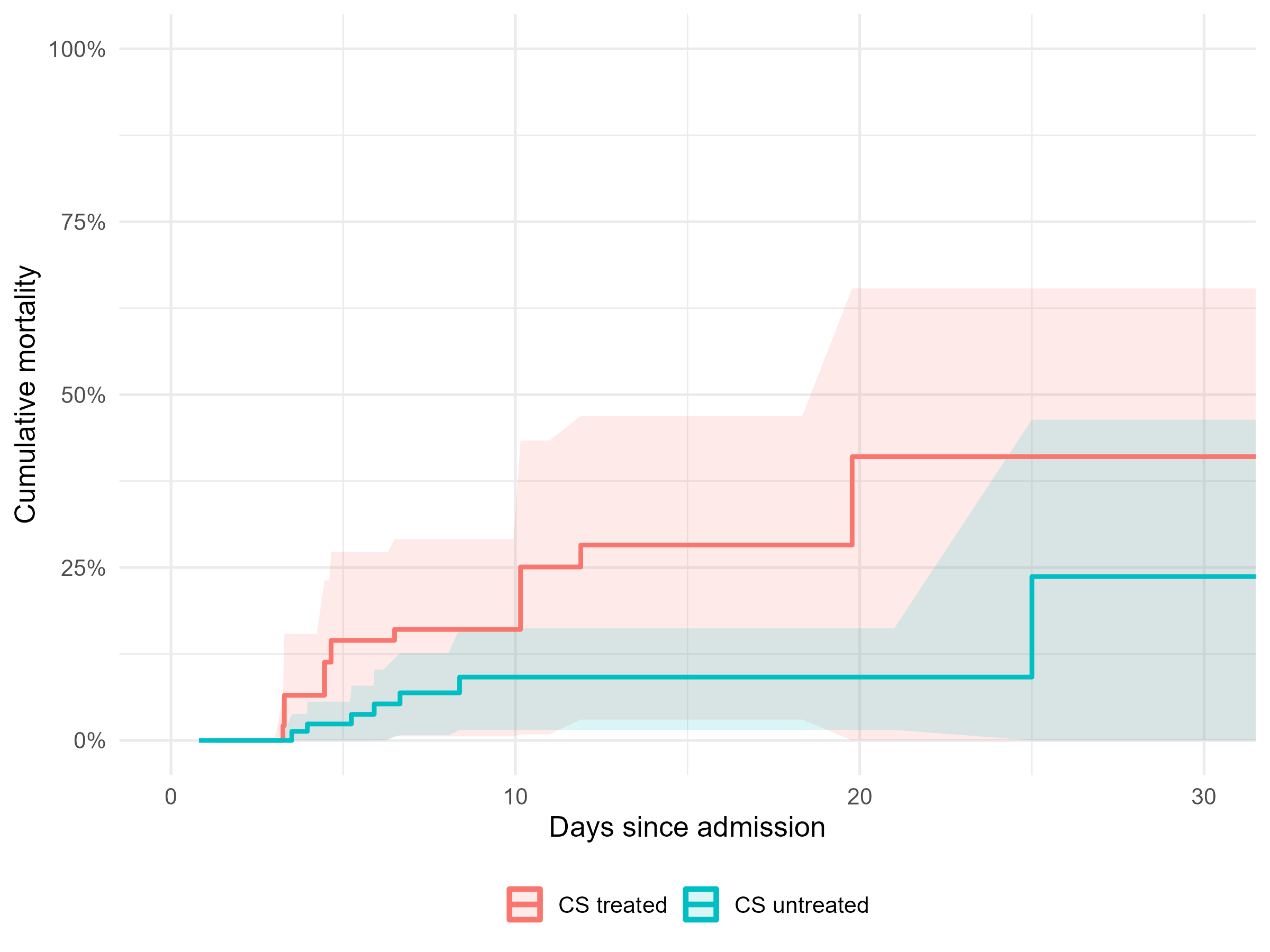

Supplement: jiaf601_Supplementary_Data [file jiaf601_supplementary_data.zip › SupplementaryFigure4.tif]
